# Detection of RINEX-2 Files With Mixed GPS L2P(Y)/L2C Carrier Phase Observations

**DOI:** 10.3390/s18124507

**Published:** 2018-12-19

**Authors:** Lambert Wanninger

**Affiliations:** Geodätisches Institut, Technische Universität Dresden, D-01062 Dresden, Germany; lambert.wanninger@tu-dresden.de

**Keywords:** GPS, L2C, quarter cycle phase shift, RINEX

## Abstract

Presently, the global positioning system (GPS) satellite constellation consists of 40% older Block IIA and IIR space vehicles and 60% newer IIR-M and IIF satellites. Only newer GPS satellites are capable of transmitting the L2C signal which is in quadrature to the legacy L2P(Y) signal being broadcast by all satellites. The data format RINEX-2 is not prepared to contain carrier phase observations of both L2 signals, but should contain either one or the other. If a mix of unaligned L2P(Y) and L2C carrier phase observations are stored in a RINEX-2 file, the quarter cycle bias causes the file to be defective and not usable for precise positioning purposes. Algorithms that detect such files are presented in this study. They are mainly based on the analysis of widelane fractional ambiguities and were applied to RINEX-2 files of 2624 reference stations. Seventy-two station files (2.7%) were found to be defective since they contained mixed and unaligned L2P(Y) and L2C carrier phase observations. If such files are used for precise positioning with ambiguities being fixed to integer values, resulting coordinate errors in long baselines can reach centimeter levels. Unaligned L2 observations often prevent ambiguity fixing, especially in short baselines.

## 1. Introduction

In 2005, the first global positioning system (GPS) Block IIR-M satellite was launched. It became the first satellite in the GPS constellation to broadcast an additional signal on L2 frequency (1227.60 MHz), the so-called L2C signal [1,2]. With this new signal, receivers had the choice to observe L2 code and carrier phases either on the legacy L2P(Y) signal, the new L2C, or both. 

**Figure 1:**
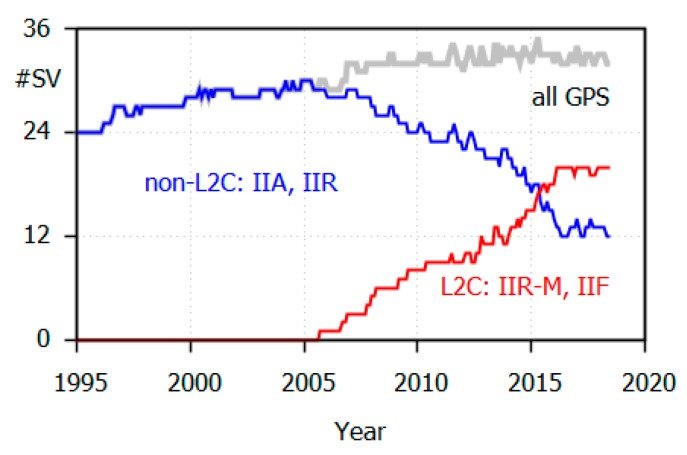
Development of the global positioning system (GPS) space segment with respect to L2C capable satellites (data source: IGS file of antenna corrections IGS14_2000.ATX).

In 2018, approximately 60% of the active GPS satellites are capable of transmitting L2P(Y) and L2C (L2C-satellites: Block IIR-M and IIF). However, there are still 12 older Block IIA and IIR (non-L2C) satellites that are restricted to L2P(Y) (Figure 1). The youngest IIR satellite was launched in 2004 and has been in operation for almost 14 years. 

The two L2 carrier components of the IIR-M and IIF satellites are fixed in phase quadrature, with L2C lagging L2P(Y) by 90 degrees [1]. Thus, a −0.25 cycle phase correction should be applied to L2C carrier phase measurement to obtain alignment [3]. If all measurements on the same frequency are aligned, any common quarter cycle phase shift is eliminated by differencing or can be estimated as a frequency-specific delay. If, however, L2P(Y) and L2C phase measurements are mixed without being aligned, severe problems in precise positioning applications occur. This mix of unaligned observations is referred to as the “L2 quarter cycle problem”.

Even if the two L2 carrier components are aligned on the level of quarter cycles, there are still satellite and receiver induced delay differences between L2P(Y) and L2C [4]. On the satellite side, they should not exceed 100 mrad [1], which equals 3.9 mm on L2. They can be determined from simultaneous L2P(Y) and L2C carrier phase observations. Berglund (2011) confirmed that the satellite induced delays are on the mm level [5]. We determined the receiver induced delay differences, and the largest differences were on the level of some mm. Since the delay differences of the aligned signals were so small, this paper only focuses on the L2 quarter cycle problem.

The RINEX-2 data format defines just one carrier phase observation on each frequency, whereas two different GPS code observations (C and P) can be stored [6]. It cannot be indicated whether carrier phase observations originate from L2P(Y) or from L2C. There is no data field that informs about any quarter cycle alignment of the carrier phase observations. The mix of L2P(Y) and L2C carrier phase observations is not considered in RINEX-2 format definition and is not explicitly prohibited. Therefore, RINEX-2 observation files are prone to containing a mix of unaligned L2P(Y) and L2C carrier phase observations.

RINEX-3 can overcome many of RINEX-2’s shortcomings [3], but for the price of more complexity, especially by defining a much larger number of observation types. Therefore, acceptance of RINEX-3 has risen only slowly, and there is still some hesitation to adopt this more complex data format. 

The next section describes the effects of mixed, unaligned L2P(Y) and L2C carrier phase observations on ambiguity estimation. Subsequently, algorithms are presented that allow the identification of RINEX-2 files being corrupted by L2 quarter cycle biases. In a further section, detection results based on the RINEX-2 observation files of more than 2600 individual stations are shown and analyzed. The last section demonstrates the effect of the L2 quarter cycle problem on coordinate estimation.

## 2. L2 Quarter-Cycle Bias and its Effect on Ambiguity Estimation

Processing carrier phase observations in baseline mode or as a single station in precise point positioning (PPP) mode requires the estimation of carrier phase ambiguities. The ambiguities of the original signals, L1 and L2, or of linear combinations of these signals, are either treated as real numbers (float solution) or fixed to their true integer values (fixed solution) [7].

In the context of the L2 quarter cycle problem, we are interested in real number L2 ambiguity estimates, and especially in fractional biases between ambiguity estimates. On L2, a GPS L2 quarter cycle bias equals 6 cm in distance. When forming the widelane linear combination WL [widelane cycles] of dual-frequency phase measurements [cycles] at frequencies 1 and 2 by:

(1) $$WL_{12}=\phi_{1}-\phi_{2},$$

we obtain a signal with a much larger wavelength (86.2 cm) that is also affected by L2 quarter cycle biases. An L2 quarter cycle bias causes a WL bias of −0.25 cy, which corresponds to −22 cm in distance and, thus, is much easier to identify. Furthermore, because of smaller residual errors from ionospheric refraction and orbits, detection of quarter cycle biases is simpler in short baselines as compared to long baselines. 

Widelane ambiguities can also be estimated using the Melbourne-Wübbena linear combination (MW). Using code measurements C [m] and carrier phase measurements [cycles] at frequencies 1 and 2, the linear combination MW [widelane cycles] is formed by:

(2) $$MW_{12}=\frac{l_{12}}{\lambda_{1}}C_{1}+\frac{l_{12}}{\lambda_{2}}C_{2}-\;\phi_{1}+\phi_{2}$$

with

(3) $$l_{12}=\frac{\lambda_{2}-\lambda_{1}}{\lambda_{1}+\lambda_{2}}$$

and $\lambda_{1},\lambda_{2}$[m] being the carrier wavelengths. This linear combination has properties of being ionosphere-free and geometry-free and, thus, it is independent of baseline length. On the other hand, it is affected by code multipath, noise, and code delays. For more information on widelane signals, see reference [8]. 

In case of undifferenced observations (PPP mode), satellite induced code and phase delays have to be corrected to enable ambiguity fixing. This is done by applying fractional cycle bias (FCB) corrections for all satellites. They can be estimated from the observation data of global networks of reference stations. As they are found to be fairly stable for the MW linear combination, a daily update seems to be sufficient to account for their temporal variations. CNES-CLS (Centre National d’Etudes Spatiales—Collecte Localisation Satellites, France) determines and publishes FCB_MW_ values under the name of widelane satellite biases (WSB) on its file transfer protocol (FTP) server [9].

FCB_MW_ values provided by CNES-CLS refer to code observations on P(Y)1 and P(Y)2. Thus, we call them *FCB_MW,P1P2_* [WL cy]. Since not all receivers observe P(Y)1, but they all observe the C/A code C1 on the first frequency, C1 and P(Y)2 are used to form MW. Thus, we have to apply P(Y)1-C1 differential code biases *DCB_P1C1_* [ns] to obtain *FCB_MW,C1P2_* [WL cy]:

(4) $$\begin{array}{ll} FCB_{MW,C1P2} & =FCB_{MW,P1P2}-\frac{f_{1}^{2}-f_{1}f_{2}}{f_{1}+f_{2}}\cdot DCB_{P1C1}\\ & \approx FCB_{MW,P1P2}-0.196\cdot DCB_{P1C1} \end{array},$$

with *f_1_,f_2_* [Hz] being the carrier frequencies. Monthly *DCB_P1C1_* values are determined and published by CODE (Center for Orbit Determination in Europe, Bern, Switzerland) [10]. Making use of these monthly values, we calculated *FCB_MW,C1P2_* and corrected the MW widelane ambiguity estimates.

After fixing and removing the widelane ambiguity, the ionosphere-free linear combination (IF) of the dual-frequency GPS carrier phase observations is obtained by:

(5) $$IF_{12}=(77\cdot \phi_{1}-60\cdot \phi_{2})\;/\;17$$

and has a wavelength of 10.7 cm [11,12]. Here, an L2 quarter cycle bias produces a bias of:

(6) $$\delta_{IF}=(-60\cdot 0.25\;cy)\;/\;17=-0.88\;cy$$

which is mainly absorbed by an ambiguity shift of one full cycle. What remains is a residual error of:

(7) $$\delta_{IF}^{\prime }=1.00\;cy-0.88\;cy=0.12\;cy\approx \;1.3\;cm.$$

Thus, the effect of an L2 quarter cycle bias on IF is much smaller than the effects on L2 or on the WL. This conclusion is true for both the ambiguity fixing (biases in units of cycles) and the coordinate solutions (biases in metric units). In conclusion, IF is not well suited for the detection of L2 quarter cycle biases, nor will coordinate solutions based on IF encounter large and thus easily detectable coordinate errors. 

**Figure 2:**
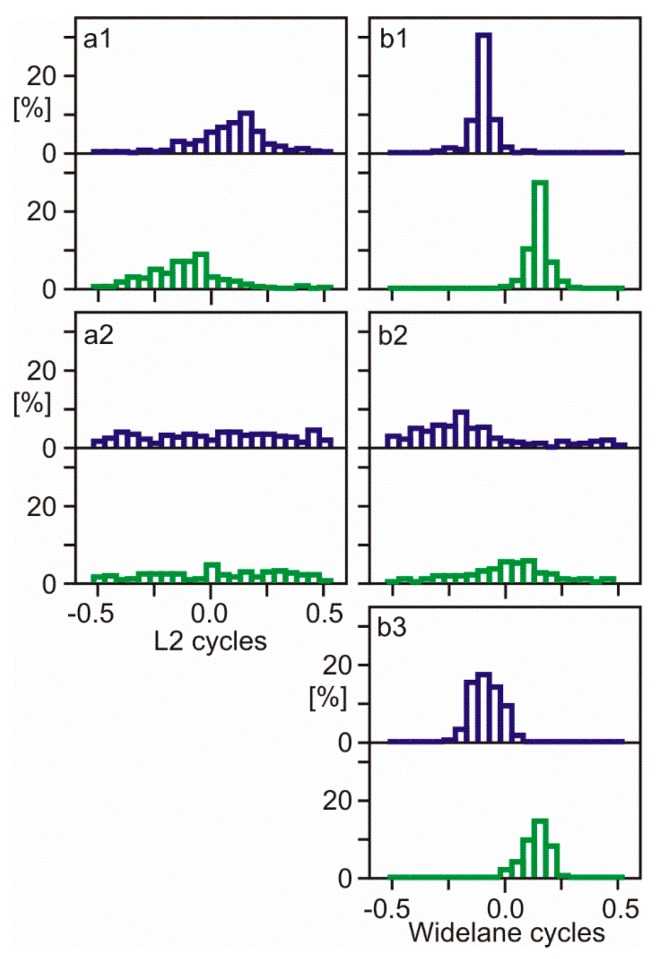
Distribution of fractional ambiguities of unaligned L2P(Y)/L2C carrier phase observations: (**a1**) L2, short baseline, (**a2**) L2, long baseline, (**b1**) WL, short baseline, (**b2**) WL, long baseline, (**b3**) Melbourne-Wübbena linear combination, PPP; each panel consists of 2 sub-panels: upper sub-panels refer to L2C, lower sub-panels refer to L2P(Y); the bin width is 0.05 cycles.

**Figure 3:**
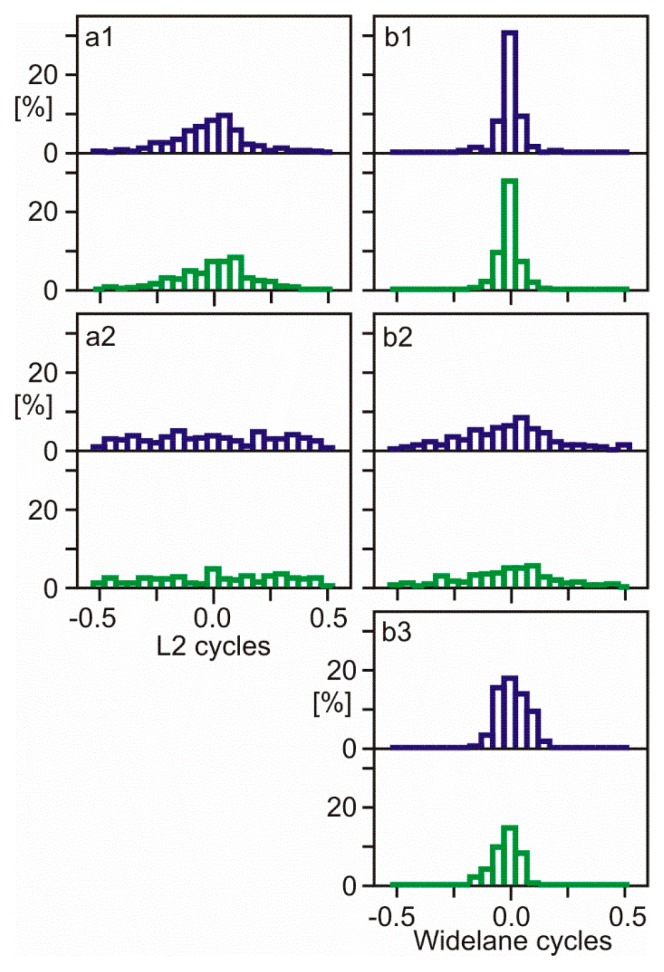
Distribution of fractional ambiguities after the alignment of L2P(Y)/L2C carrier phase observations. The same observation stations and identical figure structure as Figure 2 are used.

A following example will illustrate the effects of quarter cycle biases on estimated ambiguities. RINEX-2 observation files of the Spanish station BCNL were identified to contain mixed L2P(Y) and L2C carrier phase measurements without alignment. L2 and WL ambiguities were estimated in baselines to stations without an L2 quarter cycle problem; a baseline of 55 km to station BELL (Figure 2 and Figure 3, panels a1, b1), and a long baseline of 566 km to station AJAC (Figure 2 and Figure 3, panels a2, b2). Data processing was performed with the baseline processing engine Wa2 and used precise International GNSS Service (IGS) orbits and IGS global ionosphere maps [13]. Furthermore, PPP solutions of station BCNL were computed using software WaPPP to estimate MW widelane ambiguities (Figure 2 and Figure 3, panel b3). The IGS antenna phase center corrections were applied for satellite and receiver antennas. The elevation mask angle was set to 10 deg. 

Figure 2 shows the distribution of fractional ambiguities of unaligned L2P(Y)/L2C carrier phase observations at BCNL. The same observation dataset was re-used for Figure 3, but L2C carrier phase observations were shifted by 0.25 cycles to align all L2 phase observations. 

The L2 quarter cycle problem at BCNL can be identified by comparing the distributions of fractional ambiguities of two groups of satellites—12 satellites with L2P(Y) observations and 18 satellites with L2C (Figure 2 and Figure 3, upper and lower sub-panels). With L2 quarter cycles biases being present (Figure 2), the distribution peaks show a separation of approximately a quarter cycle. With observations being aligned (Figure 3), the distribution peaks are aligned as well. In shorter baselines with less residual ionospheric delays, L2 quarter cycle biases can be detected much better than in longer baselines. In addition, the WL shows the bias much clearer than L2. MW widelane ambiguity distribution is especially suited for quarter cycle bias detection because it is ionosphere-free and has an important additional advantage—the data processing requires only measurements of the one station to be examined.

## 3. Detection Algorithms

As shown in the previous section, L2 quarter cycle biases are detected best by analyzing fractional widelane ambiguities. In this section, three detection algorithms are presented. The first two make use of estimated widelane ambiguities, either in PPP or in baseline mode. The third algorithm compares corresponding RINEX-2 and RINEX-3 observation files.

### 3.1. Algorithm 1: Melbourne-Wübbena Linear Combination in PPP Mode

In this approach, widelane ambiguities are estimated based on the Melbourne-Wübbena (MW) linear combination in single station processing (PPP). FCB_MW_ corrections must be applied to obtain unbiased ambiguities. 

All differences between the ambiguities of one satellite are treated as cycle-slips and can usually be fixed to their true integer values and removed. Thus, we obtain one MW ambiguity estimate A_MW_ per satellite. 

The ambiguities are separated into two groups: non-L2C and L2C satellites. Within each group, the estimated ambiguities are averaged using the algorithm suggested by Gabor and Nerem (2002) [14]. In a first step, the ambiguity angles are transformed to vectors applying sine and cosine functions. These functions also remove the integer portions of the ambiguities. Summing these vectors creates the two components of a resulting vector:

(8) $$\begin{array}{l} \Sigma_{\sin}=\sum sin\;\left(2\pi \cdot A_{MW}\right)\\ \Sigma_{\cos}=\sum cos\;\left(2\pi \cdot A_{MW}\right) \end{array}.$$

The average fractional ambiguity *AFA_MW_* is then recovered by:

(9) $$AFA_{MW}=\frac{1}{2\pi }arctan2\;\left(\Sigma_{\sin},\Sigma_{\cos}\right),$$

where the *arctan*2 function ensures the correct quadrant. This function is available with many programming languages. 

Since we are also interested in quality measures of these average values, residuals are computed for all ambiguities by:

(10) $$R_{MW}=A_{MW}^{}-AFA_{MW}^{}$$

and fractional residuals *FR_MW_* are obtained by removing any integer portions by:

(11) $$FR_{MW}=\frac{1}{2\pi }arctan2\;\left(\sin\;\left[2\pi \;R_{MW}\right]\;,\cos\;\left[2\pi \;R_{MW}\right]\right).$$

The L2 bias between non-L2C and L2C satellites is the difference:

(12) $$b_{MW}=AFA_{MW}^{L2C}-AFA_{MW}^{non-L2C}.$$

Its standard deviation is computed from the fractional residuals of the two groups of satellites:

(13) $$s_{b}=\sqrt{\frac{\sum {\left(FR_{MW}^{non-L2C}\right)}^{\;2}}{n\cdot (n-1)}+\frac{\sum {\left(FR_{MW}^{L2C}\right)}^{\;2}}{m\cdot (m-1)}},$$

where *n* is the number of ambiguities of non-L2C satellites and m the number of ambiguities of L2C satellites.

In a few cases, this detection algorithm produced dubious results with large standard deviations. The detailed analysis revealed that FCB corrections, which work well for the majority of stations, fail with some data sets. In these cases, alternative detection algorithms are needed.

### 3.2. Algorithm 2: Carrier Phase Widelane in Baseline Mode

This algorithm uses widelane fractional ambiguities of the carrier phase WL linear combination (1) and is not influenced by any biases affecting code measurements. In order to keep residual ionospheric effects small, IGS global ionosphere maps are used to reduce ionospheric delays. Since remaining ionospheric errors are still too large, this detection algorithm is used in baseline mode, reducing residual ionospheric delays further by differencing. While working in baseline mode, which is only able to determine differences in L2 biases, it is essential to know whether the second station is affected by the L2 quarter cycle problem. 

The WL linear combination is influenced by orbit errors, which makes it necessary to use precise IGS orbits. Also, tropospheric zenith delays need to be estimated. The analysis of fractional ambiguities is done similarly to Algorithm 1. Experiences show that Algorithm 2 produces reliable results for baseline lengths of up to several 100 km. If, however, large residual ionospheric errors exist, no definite results are obtained. 

### 3.3. Algorithm 3: Comparison with RINEX-3 Observation File

In the cases where RINEX-2 and corresponding RINEX-3 observation files are both available, one-to-one comparison of measurements reveals whether the RINEX-2 file contains mixed L2 phase observations. In addition, comparing simultaneous RINEX-3 carrier phase measurements on the same frequency reveals whether phase alignment was performed.

In the example of Table 1, the RINEX-2 file contains GPS carrier phase observations on three frequencies: L1, L2, and L5. The corresponding RINEX-3 file lists four different GPS phase observations: L1C, L2S, L2W, and L5Q. On GPS frequencies one and five, the numerical values are identical or differ by 0.001 cy maximum. Any matching of observation codes is trivial: L1–L1C, L5–L5Q. On the second frequency, the RINEX-3 file contains phase observations obtained in two different tracking modes: L2W (L2P(Y)) and L2S (L2C, CM code). L2W observations are available for all satellites, L2S for Block IIR-M and IIF only. When L2W and L2S are both available, their numerical values differ by a few full cycles and some hundredth of a cycle, but not by a quarter cycle. Thus, these two observations are aligned in RINEX-3, which is also indicated by the “PHASE SHIFT” information in the header section of the file. For non-L2C satellites, L2 observation values are identical to L2W, or they differ by 0.001 cy maximum. For all other satellites, full cycles of L2 are identical to L2S, but not to L2W. However, there is a quarter cycle phase shift between L2 and L2S, which means that the alignment of L2S was not performed for L2. The conclusion is that the L2 carrier phase observations are a mix of L2P(Y) and L2C. Since they are not aligned, the L2 quarter cycle problem exists in this RINEX-2 file.

## 4. Observation Data Sets and Detection Results

The detection algorithms described above were applied to RINEX-2 observation files of DoY (day of year) 150, 2018, which were copied from the servers of four GNSS data centers (Table 2). The data center operated by Crustal Dynamics Data Information System (CDDIS) contains mainly, but not exclusively, observations of stations of the IGS. Geoscience Australia manages a database of GNSS reference stations for the realization of the Asia-Pacific Reference Frame (APREF). The German Federal Agency for Cartography and Geodesy (BKG) serves as one of the data centers of the EUREF Permanent Network (EPN). The US National Geodetic Survey (NGS) maintains a database of Continuously Operating Reference Stations (CORS) in the USA and some additional countries. 

Some stations provide their RINEX-2 files to more than one of the four data centers of Table 2. We deleted such redundant files, keeping just one copy—the one obtained from CDDIS.

CDDIS, APREF, and EPN/BKG offer RINEX-2 and RINEX-3 observation files. Usually, if a RINEX-3 file is available, the corresponding RINEX-2 file is also present. NGS provides observations in RINEX-2 only.

**Figure 4:**
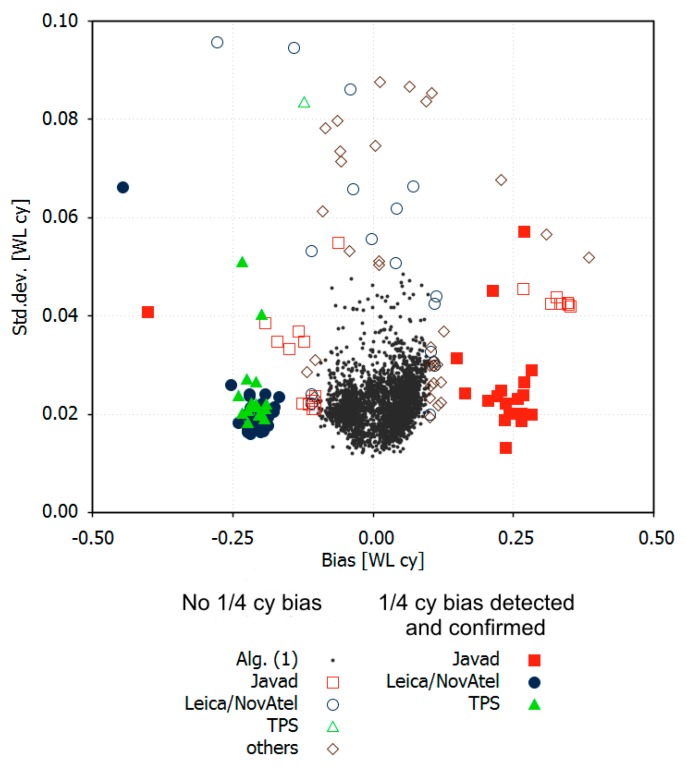
Estimated L2 biases between non-L2C and L2C measurements according to algorithm 1, validations (filled symbols) or rejections (open symbols) by Algorithms 2 or 3.

Detection Algorithm 1 was applied to all 2624 station files. The primary results consisted of the bias difference between non-L2C and L2C satellites according to (12) and its standard deviation according to (13). Of the stations, 94.6% had a bias smaller than 0.1 cycles with a standard deviation of less than 0.05 cycles. They were considered to be correct and not affected by the L2 quarter cycle problem (black dots in Figure 4). The remaining 143 observation files were treated as suspicious and checked by Algorithms 2 and 3. Algorithm 2 was used when the closest GPS station was not further apart as several 100 km. Algorithm 3 was applied if a corresponding RINEX-3 file was available. The number of detected and confirmed RINEX-2 files with L2 quarter cycle problems amounted to 72, which was 2.7% of all examined files (filled colored symbols in Figure 4). L2 carrier phase observations of the other 71 station files exhibited no L2 quarter cycle problems (open symbols in Figure 4). Some of them showed large systematic biases between L2C and non-L2C satellites, which cannot be explained by phase biases. Systematic delays of the code signals are suspected to be the main cause.

The validation by Algorithms 2 and 3 reveals that Algorithm 1 produces reliable detection results if the absolute value of the computed bias is larger 0.125 WL cy and its standard deviation is smaller than 0.03 WL cy. If the standard deviation is larger, correct and defective RINEX-2 files cannot always be reliably separated using Algorithm 1. Then, the other algorithms must also be applied, which considerably increases the necessary effort.

The data centers of CDDIS, EPN/BKG, and NGS contain RINEX-2 files affected by the L2 quarter cycle problem, whereas the Australian data center, APREF, does not (Table 3). This is true for those files that are available at APREF only. As mentioned before, files present at CDDIS and other data centers were assigned to CDDIS and the affected files are listed for this data center. In fact, even APREF contains a few affected RINEX-2 files, which were copied from other data centers.

**Figure 5:**
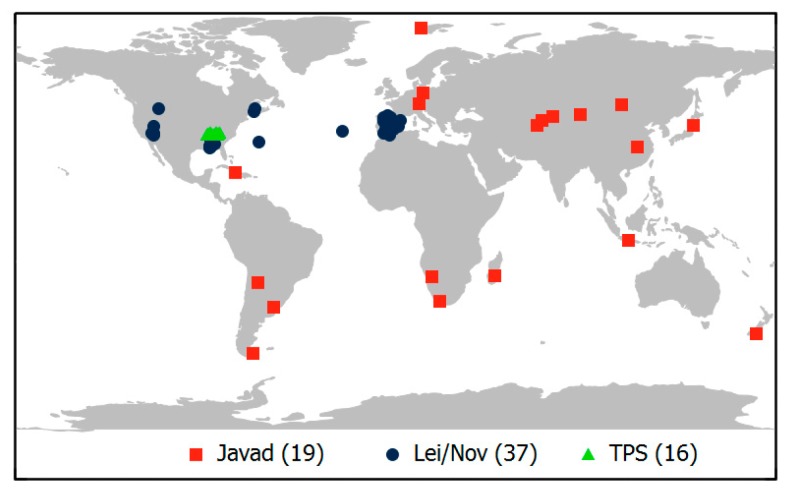
Geographical distribution of stations providing RINEX-2 files with L2 quarter cycle problems.

Within the networks of EPN/BKG and NGS, most affected stations are geographically clustered: Spain, Tennessee, Alabama, and Nevada (Figure 5). In both networks, station operators are in charge of certain regions, and there they tend to have the same receiver configuration and software setups. Most affected stations files from CDDIS belong to a single agency that operates receivers from one manufacturer and probably uses similar software setups.

All affected stations are equipped with receivers from just a small number of manufacturers. The number of receiver manufacturers without any affected stations is much longer (Table 4). It should be emphasized that the L2 quarter cycle problem is not expected to be caused by faulty receivers but rather by inadequate receiver configuration or, probably more often, by using incorrect option settings of the RINEX-2 conversion software. 

Affected stations with TPS or Leica/NovAtel receivers exhibit a MW bias of around −0.25 cycles, whereas most Javad receivers show quarter cycle biases of the opposite sign (Figure 4). This is in accordance with earlier findings by Wübbena et al. (2012) [15] and indicates that the RINEX-2 L2 quarter cycle problem has probably existed for many years and is a persistent one. 

Independent of the L2 quarter cycle problem, the results illustrated by Figure 4 contain valuable information on the ability to fix MW ambiguities in PPP data processing. Small standard deviations indicate that the applied *FCB_MW,C1P2_* corrections are valid for this observation data set, and MW ambiguity fixing is not affected by any biases. Of all the examined station files, 97.3% produced very small standard deviations of less than 0.04 WL cy. The remaining 2.7%, however, had larger standard deviations, some even exceeding 0.08 WL cy. Here, remaining code delays can prevent successful widelane ambiguity fixing.

## 5. Coordinate Errors Due to Quarter Cycle L2 Biases

If carrier phase ambiguities are estimated as real values and not fixed to integer values (float solution), the quarter cycle L2 bias does not cause any effects to coordinate solutions since it is completely absorbed by the real value ambiguities. However, after fixing ambiguities to integer values, the biases cause coordinate errors. The size of the coordinate errors depends on a large number of factors, among which are the ratio of non-L2C and L2C GPS satellites, the completeness of ambiguity fixing, the geometry of satellite-receiver constellation, the elevation mask angle, the observation weighting, the length of static observation session, and the kind of coordinate solution.

Experiences with our own software modules, Wa2 and WaPPP, show that on short baselines, the L2 quarter cycle problem leads to an incomplete fixing of ambiguities. On long baselines or with PPP, however, the algorithms accept larger residual ionospheric effects or larger biases in general, which enables complete ambiguity fixing. 

In order to be able to estimate the size of the coordinate errors, at least for certain positioning modes, zero-baseline tests were performed. Two observation datasets were selected which form a zero-baseline:

1. an original observation data set or a simulated data set, and
2. a duplicate of data set (1) with quarter cycle L2 biases introduced/removed for all L2C carrier phase observations, while L2P(Y) carrier phase observations remained unchanged.

Since the quarter cycle biases are the only difference between the two data sets, only they affect the differential coordinate solution. All other error sources, such as the effects caused by the ionosphere, troposphere, orbit errors, multipath, etc., cancel out in the baseline processing since they are completely identical in corresponding data sets 1 and 2. We altered the global navigation satellite system (GNSS) baseline processor Wa2 in such a way that the fixing algorithms intended for long baselines (WL, IF) could also be used for these zero-baselines. A complete ambiguity fixing could always be achieved.

The coordinate solution after ambiguity fixing can be computed in several different ways. All results shown in this section are based on the IF linear combination of carrier phase observations and the estimation of tropospheric zenith delays being enabled. This setting simulates long baselines or PPP solutions with all ambiguities being fixed. According to Equation (7), when using the IF linear combination, the largest portion of an L2 quarter cycle bias is absorbed by an incorrect fixing of the ambiguity by 1 cycle. What remains is a phase bias in the order of just 1 cm. 

**Figure 6:**
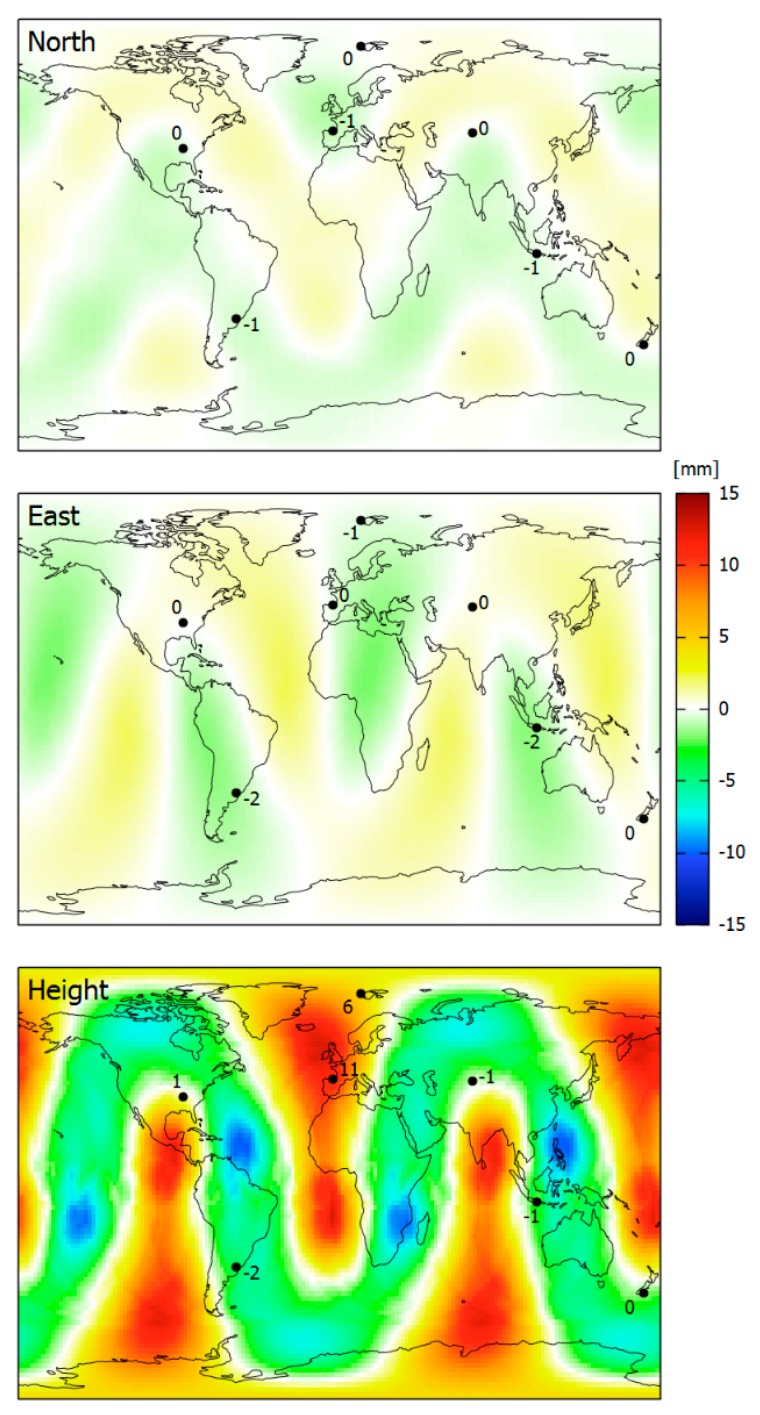
Coordinate errors of 24 h static positioning in early 2018 due to L2 quarter cycle biases: ionosphere-free linear combination with ambiguities being fixed, estimation of tropospheric zenith delays enabled, based on simulated stations in a 2° × 2° grid and on seven real stations.

A first example shows the geographical distribution of coordinate errors for long-term (24 h) static observations (Figure 6). It is mainly based on simulated data, computed for DoY 150, 2018 with an elevation mask of 5 degrees. The simulation was performed for 16,380 stations in a regular grid with 2° × 2° spacing. In order to confirm the results, seven real stations were selected from the data set of the last section, and a similar zero-baseline computation was performed with their RINEX-2 observation data. 

The coordinate errors reach up to 1.3 mm, 2.1 mm, and 12.6 mm in the north, east, and height components, respectively. There is a distinct geographical distribution with areas of larger coordinate errors and others with small ones (Figure 6).

The results based on simulated observations match very well with those of the real datasets. Any differences are due to missing observations in the real datasets above the 5 deg elevation mask angle. Observation errors are not able to affect the solutions since they cancel out by forming zero-baselines. 

**Figure 7:**
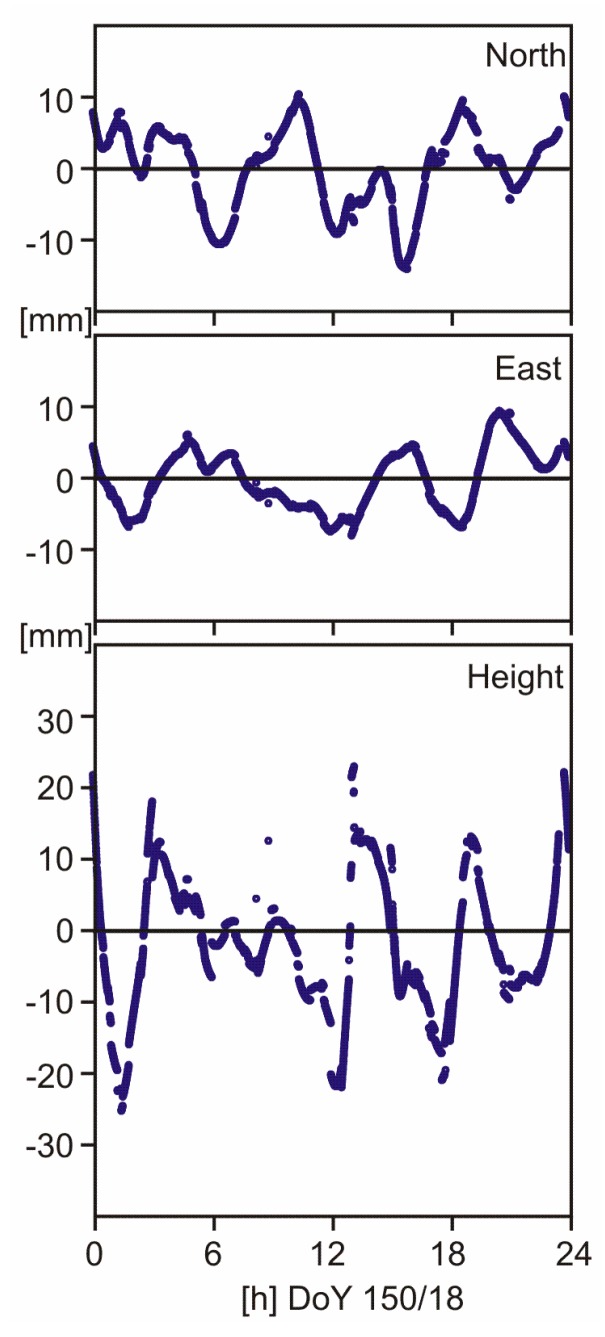
Epoch-by-epoch coordinate errors of station BIK0 (Bishkek, Kyrgyzstan) on DoY 150/18 due to L2 quarter cycle biases: ionosphere-free linear combination with ambiguities being fixed, estimation of tropospheric zenith delays enabled.

One of the real data sets belongs to station BIK0 (Bishkek, Kyrgyzstan). Here, daily coordinate errors are smaller than 1 mm in all three coordinate components. We picked this one to show the epoch-wise variations of coordinate errors (Figure 7). They reach centimeter level in north and east, and the level of a few centimeters in the height component. Jumps in the time series are due to changes in satellite constellation caused by rising or setting satellites or by missing observations above the elevation mask angle of 5 deg.

Due to the daily repetition of the GPS satellite orbits, the described geographical distribution of daily coordinate errors is stable as long as the GPS satellite constellation remains unchanged. In the near future, we will experience replacements of older GPS satellites by Block III satellites, which will enlarge the already existing majority of L2C-capable space vehicles. This will reduce the coordinate errors produced by defective RINEX-2 files. 

## 6. Conclusions and Outlook

The detection of mixed GPS L2P(Y)/L2C carrier phase observations in RINEX-2 files with non-aligned quarter cycle biases is performed best by analyzing fractional widelane ambiguities. Either of the MW linear combination or of the widelane carrier phase linear combination. The main advantage of an analysis in PPP mode lies in the simplicity of the processing setup since no baselines need to be formed. Its drawback comes from the dependence of the MW linear combination on code delays. Some receivers seem to possess MW code delays that do not fit to the satellite individual fractional bias corrections valid for the majority of the receivers. 

If mixed carrier phase observations are not aligned on the level of quarter cycles, the L2 bias can prevent ambiguity fixing. However, ambiguity fixing is often successful despite an L2 bias in long baselines or in PPP mode where fixing algorithms based on widelane and ionosphere-free linear combinations are used. Coordinate errors that reach the centimeter level occur with the GPS satellite constellation of early 2018, even in daily solutions, and mainly affect the height component. These coordinate errors depend on the geographical location of the station. There are regions where the coordinate errors average out and do not exceed 1 mm over a period of one day. 

The L2 quarter cycle problem can easily be resolved by carefully selected settings of GNSS receiver and RINEX conversion software. It would also disappear with the complete transition from data format RINEX-2 to RINEX-3. In a few years’ time, all Block IIA and IIR satellites will have ceased operation. Furthermore, the US government restricted its commitment to support GPS P(Y) signals until at least two years after there are 24 operational satellites broadcasting L5, i.e., 24 satellites of Block IIF and III [16]. When P(Y) code signals are not available anymore, the L2 quarter cycle problem vanishes. On the other hand, with the introduction of L1C on Block III satellites [1,17], a new quarter cycle problem may occur in RINEX-2 files since L1C and the legacy L1C/A signal will be in quadrature.

The data centers or station operators were informed about their stations with detected L2 quarter cycle problems. Receiver configuration or RINEX conversion software settings of several of the affected stations were adapted so that these stations do not produce any further defective RINEX-2 files.

## Figures and Tables

**Table 1:** Two sample data sets of corresponding RINEX-2 and RINEX-3 phase observations [cy] used to map RINEX-3 onto RINEX-2 observation codes. EUREF Permanent Network station CARG, day of year (DoY) 150, 2018, epoch 00:00:00.

Satellite	RINEX-2	RINEX-3
	carg1500.18o	CARG00ESP_R_20181500000_01D_30S_MO.rnx
PRN 01 (Block IIF, L2C)	L1: 130395895.177 L2: 101607188.874 L5: 97373552.985	L1C: 130395895.176 L2W: 101607184.615, L2S: 101607188.624 L5Q: 97373552.984
PRN 02 (Block IIR, non-L2C)	L1: 128919863.442 L2: 100457022.515 L5: -	L1C: 128919863.442 L2W: 100457022.516, L2S: - L5Q: -

**Table 2:** GPS observation data sets, RINEX-2, DoY 150, 2018.

Data Center	Geographical Distribution of Stations	Access
CDDIS	Global	ftp://cddis.gsfc.nasa.gov/pub/gps/data/daily/2018/150/18d
APREF	Mainly Australia/New Zealand	ftp://ftp.ga.gov.au/geodesy-outgoing/gnss/data/daily/2018/18150
EPN/BKG	Europe	ftp://igs.bkg.bund.de/EUREF/obs/2018/150
NGS	Mainly USA	ftp://geodesy.noaa.gov/cors/rinex/2018/150

**Table 3:** Detection Results by Data Center.

Data Center	Number of Station Files Processed	Number of RINEX-2 Files with L2 Quarter Cycle Problem
CDDIS	495	23 (4.6%)
APREF	186	-
EPN/BKG	223	13 (5.8%)
NGS	1720	36 (2.1%)
Sum	2624	72 (2.7%)

**Table 4:** Detection Results by Receiver Manufacturer.

Receiver Manufacturer	Number of Station Files Processed	Number of RINEX-2 Files with L2 Quarter Cycle Problem
AOA	1	-
Ashtech	34	-
CHC	2	-
ITT	15	-
Javad	110	19 (17.3%)
JPS	9	-
Leica/NovAtel	686	37 (5.4%)
Septentrio	168	-
TPS	111	16 (14.4%)
Trimble/Champion	1488	-
Sum	2624	72 (2.7%)
